# Survival Outcomes of BCG Only, BCG Plus EMDA-MMC or Upfront Radical Cystectomy in High-Risk Non-Muscle Invasive Bladder Cancers (NMIBCs): A Multicentre, International, Collaborative Study from Tertiary Referral Institutions

**DOI:** 10.3390/cancers18030500

**Published:** 2026-02-03

**Authors:** Francesco Del Giudice, Valerio Santarelli, Amir Khan, Mohamed Gad, Katarina Spurna, Syed Ghazi Ali Kirmani, Noor Huda Bhatti, Rajesh Nair, Kathryn Chatterton, Suzanne Amery, Elsie Mensah, Benjamin Challacombe, Youssef Ibrahim, Felice Crocetto, Giuseppe Basile, Roberta Corvino, Eleonora Razeto, Matilde Verde, Vincenzo Asero, Ettore De Berardinis, Giulio Garaffa, Jan Łaszkiewicz, Aleksander Ślusarczyk, Francesco Claps, Benjamin I. Chung, Ramesh Thuraraja, Timothy O’Brien, Muhammad Shamim Khan, Yasmin Abu-Ghanem

**Affiliations:** 1 Department of Maternal-Infant and Urological Sciences, “Sapienza” University of Rome, Umberto I Hospital, 00185 Rome, Italy; 2 Department of Urology, Stanford University School of Medicine, Stanford, Palo Alto, CA 94305, USA; 3 Guy’s and St. Thomas’ NHS Foundation Trust, Guy’s Hospital, London SE1 9RT, UK; 4 Division of Urology, Department of Surgery, University of Maryland, Baltimore, MD 21201, USA; 5 Department of Neurosciences, Reproductive Sciences and Odontostomatology, University of Naples “Federico II”, 80131 Naples, Italy; 6 Department of Urology, IRCCS Ospedale San Raffaele, Vita-Salute San Raffaele University, 20132 Milan, Italy; 7 The Institute of Urology, University College London Hospitals, London NW1 2BU, UK; 8 Department of Minimally Invasive and Robotic Urology, University Center of Excellence in Urology, Wroclaw Medical University, 50-556 Wroclaw, Poland; 9 Department of General, Oncological and Functional Urology, Medical University of Warsaw, 02-091 Warsaw, Poland; 10 Urological Clinic, Department of Medicine, Surgery and Health Sciences, University of Trieste, 34149 Trieste, Italy

**Keywords:** non-muscle-invasive bladder cancer (NMIBC), transurethral resection of bladder tumour (TURBT), radical cystectomy (RC), Bacillus Calmette–Guerin (BCG), bladder cancer (BC)

## Abstract

**Introduction**: Conservative or upfront radical management for high- and very high-risk non-muscle-invasive bladder cancer continues to be debated, particularly for cases with adverse pathological features. We aimed to compare survival outcomes among NMIBC patients treated with transurethral resection of bladder tumour (TURBT) followed by either Bacillus Calmette–Guérin (BCG), sequential BCG plus electromotive administration of mitomycin C (EMDA-MMC), or upfront radical cystectomy (RC). **Materials and Methods**: High- and- very high-risk NMIBC cases undergoing TURBT followed by BCG, BCG plus EMDA-MMC, or RC at two international tertiary referral centres between 2009 and 2024 were retrospectively reviewed. Recurrence-free survival (RFS), progression-free survival (PFS), and overall survival (OS) were estimated using Kaplan–Meier methods. Multivariable Cox regression models were applied to identify factors independently associated with survival outcomes. **Results**: A total of 1178 patients were included: 852 received BCG, 249 received BCG/EMDA-MMC, and 77 underwent upfront RC. Kaplan–Meier analysis revealed no significant differences in RFS or PFS between the BCG and BCG/EMDA-MMC groups, nor in OS between the three treatment strategies. According to multivariable analysis, concomitant carcinoma in situ (CIS) and increasing T stage at TURBT were independently associated with poorer RFS (HR 1.39; 95% CI 1.05–1.85), PFS (HR 1.95; 95% CI 1.36–2.82), and OS (HR 2.28; 95% CI 1.60–3.25). A second resection conferred a protective effect on PFS (HR 0.72; 95% CI 0.54–0.95). Treatment modality (BCG, BCG/EMDA-MMC, or upfront RC) was not significantly associated with any survival endpoint. **Conclusions**: In this large multicentre series of patients with high- and very high-risk NMIBC undergoing TURBT, survival outcomes were primarily influenced by clinical–pathological characteristics rather than the adjuvant treatment of choice.

## Simple Summary

In patients with high- and very high-risk Non-muscle-invasive Bladder Cancer, the risk of recurrence and progression remains high despite standard therapies. This multicentre study compared three adjuvant treatment strategies: BCG, BCG combined with electromotive administration of mitomycin C, and upfront radical cystectomy. The results showed no significant differences in survival outcomes among the treatment approaches. Instead, patient- and tumour-related factors, such as tumour stage, presence of concomitant carcinoma in situ, and repeat transurethral resection, had a greater impact on clinical outcomes. These findings suggest that treatment decisions should be individualized based on disease characteristics and patients’ preferences balanced with oncologic risk and clinical judgement.

## 1. Introduction

Bladder cancer (BC) is the 10th most common cancer worldwide, and the 6th in men, with an age-standardized incidence rate of approximately 11 per 100,000 in both Europe and North America [1]. Non-muscle-invasive BC (NMIBC) accounts for approximately 75% of new BC diagnoses [2]. While NMIBC generally follows a more indolent clinical course compared to Muscle-Invasive disease, it is associated with a particularly high recurrence rate. As a consequence, NMIBC has a disproportionately high prevalence relative to its incidence and imposes a substantial burden on healthcare systems due to the need for lifelong surveillance, repeated endoscopic procedures, and prolonged intravesical treatments [1,2]. Despite its more indolent status at diagnosis, NMIBC carries a clinically meaningful risk of disease progression. Indeed, up to 45% of high-grade NMIBCs progress to muscle-invasive BC (MIBC) within 5 years of initial diagnosis [3]. The risk of progression is not uniform across all patients but depends on a range of tumour- and patient-related variables. These include the number of lesions, pathological T stage, tumour size, histological grade, presence of concomitant CIS, whether the tumour is primary or recurrent, and the timing of recurrence, particularly within the first year following initial treatment [3,4]. A major focus of research in the field has been the establishment of an accurate risk stratification system, able to precisely predict the individual likelihood of progression to MIBC [5]. Accordingly, such risk classification should influence the aggressiveness of management choices, ranging from fulguration or transurethral resection of bladder tumour (TURBT) alone, to TURBT followed by adjuvant intravesical chemotherapy or immunotherapy and, in selected cases, upfront radical cystectomy (RC) [6,7]. In view of expanding evidence and an increasingly complex therapeutic landscape, current European Association of Urology (EAU) guidelines have adopted a four-class risk stratification system, categorizing patients into low-, intermediate-, high-, and very high-risk NMIBC [8]. This novel risk stratification, taking into account patient and tumour characteristics, as well as both the WHO 1973 and WHO 2004/2016 grading systems, aims to predict the risk of progression to MIBC with good accuracy. Based on this model, the estimated probability of progression at 1, 5, and 10 years can be as low as 0.06%, 0.57%, and 3% for low-risk NMIBC, respectively, and as high as 20%, 44%, and 59% for very high-risk MIBC, respectively [8]. TURBT alone or followed by a single immediate post-TURBT chemotherapy instillation is the recommended approach for low-risk NMIBC. Intermediate-risk BC should be managed with TURBT followed by one year of adjuvant chemotherapy or Bacillus Calmette-Guérin (BCG) therapy. Finally, a 3-year scheme of BCG or upfront radical cystectomy are the recommended management options of high-risk and very high-risk NMIBC [9]. Particularly for the very high-risk group, immediate RC is strongly advised by current EAU guidelines [10]. Nonetheless, RC is associated with high rates of procedure-related complications and readmissions. In addition, the impact of RC on urinary and sexual function, as well as on body image, leads to a significant decrease in perceived quality of life (QoL) [4,10]. To further reduce the risk of recurrence and progression, and to avoid a more radical management option such as upfront RC, Di Stasi and colleagues, nearly 20 years ago, proposed a sequential combination of mitomycin C (MMC) with electromotive drug administration (EMDA) with BCG [11]. The efficacy of this combination, also known as the BCG/EMDA-MMC protocol, has been demonstrated in a small randomized clinical trial (RCT) [11]. Given the absence of confirmatory data, the BCG/EMDA-MMC protocol is not presently incorporated into NMIBC management algorithms [12,13]. Moreover, the additional procedural complexity compared to standard intravesical therapy and the need for specialized equipment limit the widespread adoption of this technology.

The aim of this multicentre study is to compare preoperative patient and tumour characteristics, as well as long-term survival outcomes, of a cohort of high-risk and very high-risk NMIBC patients managed with TURBT followed by adjuvant BCG alone, BCG in combination with EMDA-MMC (i.e., BCG/EMDA-MCC), or upfront RC at two large international tertiary referral centres for BC. Ultimately, this study seeks to provide additional evidence to inform risk-adapted, individualized management of high-risk and very high-risk NMIBC.

## 2. Materials and Methods

### 2.1. Study Cohort

Patients with high-risk and very high-risk NMIBCs treated from 2009 to 2024 in the two high-volume referral centres were retrospectively reviewed. Preoperative patient and tumour characteristics were recorded (age, gender, stage, grade, and risk class). Routinely, BC cases were discussed in urothelial multi-disciplinary team meetings (MDT) prior to management proposal following EAU guidelines in force at the time of treatment, or according to IRB-approved practice, in line with the Declaration of Helsinki and Good Clinical Practice (GCP) guidelines. Inclusion criteria were histologically confirmed diagnosis of urothelial or mixed-histology primary BC; high-risk and/or very high-risk group; absence of distant metastases at preoperative clinical staging; BC managed with TURBT +/− a second resection, followed by adequate BCG exposure (defined as the completion of at least five of six doses of an initial induction course plus at least two out of six doses of a second induction course or two out of three doses of maintenance therapy), or BCG/EMDA-MCC protocol, or with upfront RC; and deemed able to make informed decisions regarding the management plan and to sign indicating informed consent for the procedures, adjuvant therapies, and the adoption of de-identified data for research purposes. Specifically, among those undergoing RC, only patients with pathologically confirmed NMIBC or pT0 status were included. Those with low- and intermediate-risk NMIBC, MIBC, metastatic BC, or TURBTs and RC performed for other indications rather than high-risk and very high-risk NMIBC were not included. As per appropriate guidelines, preoperative local and distant staging was performed with a combination of TURBT, multiparametric magnetic resonance imaging (mpMRI), computerized tomography (CT), and fluorodeoxyglucose positron emission tomography/CT (PET CT), following MDT recommendations and in accordance with indications and contraindications for each diagnostic modality.

### 2.2. Patient Management

All TURBTs and RCs were performed by a team of consultants, experts in the management of urothelial malignancies. A second resection was performed 2–6 weeks after the initial white-light TURBT in patients with clear residual disease, all T1 tumours, and all cases with an absent detrusor muscle at initial histological specimen collection. RCs were performed with either a robot-assisted or open approach, and were followed by urinary diversion with either ileal conduit (IC) or orthotopic neobladder. A pelvic lymphadenectomy (PLND) was performed in selected cases, with either standard or extended template, according to the surgeon’s preference and MDT recommendation. RC and TURBT specimen evaluation were carried out by experienced uropathologists of the respective institution. BCG therapy was administered following the initial Morales et al. protocol with later refinements by the Southwestern Oncology Group (SWOG) trials, in a 6-week induction phase followed by maintenance scheme [14,15]. Sequential BCG and EMDA-MCC were combined as per the Di Stasi protocol originally described [11]. In summary, it involved a sequential course of two weekly BCG instillations followed by one weekly EMDA-MMC instillation (40 mg mitomycin C delivered under a 20 mA electric current for 30 min), repeated for three consecutive cycles (i.e., induction course). Maintenance therapy consisted of alternating EMDA-MMC and BCG monthly for three cycles in the sequential arm. All cases treated with BCG/EMDA-MMC or upfront RC were retrospectively reviewed from the prospectively maintained Guy’s and St Thomas’ Hospital (London, UK) database, whereas the BCG-only cohort was entirely derived from the Department of Maternal Infant and Urologic Sciences, “Sapienza” University of Rome (Rome, Italy).

### 2.3. Statistical Analysis

Statistical analysis was performed with Stata version 18.1 (Stata Corporation, College Station, TX, USA) and SPSS version 27.0 (IBM SPSS Statistics for Windows, Version 27.0. Armonk, NY, USA: IBM Corp.), in accordance with previously described methodology and following separate analytic steps [16]. Firstly, pertinent study information was summarized using descriptive statistics and stratified according to treatment group (BCG alone vs. BCG plus EMDA-MMC vs. upfront RC). Median with interquartile range (IQR) was adopted to report continuous variables, and the numerosity of the samples for categorical variables was expressed as numbers and percentages. Fisher’s exact test or the Pearson Chi-square test was adopted to test the association between variables. Pairwise intergroup comparison of variables and quantitative data analysis was performed with the Mann–Whitney test or ANOVA one-way test. Secondly, Kaplan–Meier survival analysis with the log-rank and generalised Wilcoxon (Breslow) or Tarone–Ware tests, when appropriate, were performed to depict the univariate impact of the treatment group on relevant continuous survival measures (recurrence-free survival [RFS], progression-free survival [PFS], and overall survival [OS]). Finally, multivariate Cox regression models estimating the independent impact of selected variables on RFS, PFS, and OS were performed.

## 3. Results

### 3.1. Study Cohort Characteristics According to the Treatment Group

Baseline characteristics of the entire population and stratified by treatment group are shown in Table 1. Median age [71 years (IQR 64–78)] and gender distribution (*n* = 976 [82.8%] males and *n* = 202 [17.1%] females) did not significantly differ between treatment groups (*p* = 0.21 and *p* = 0.22, respectively). Patients undergoing upfront RC had more frequently a higher tumour stage (*n* = 62 [80.5%] pT1 and *n* = 15 [19.5%] pTa) compared to the BCG/EMDA-MMC (*n* = 136 [54.6%] pT1 and *n* = 113 [45.4%] pTa) and BCG only (*n* = 590 [69.2%] pT1 and *n* = 262 [30.8%] pTa) groups (*p* < 0.001). The BCG/EMDA-MMC group was the group with the highest rates of low-grade tumours (*n* = 23 [9.2%] vs. *n* = 23 [2.7%] BCG and *n* = 2 [2.6%] RC, *p* < 0.001). Conversely, the BCG-only cohort had the lowest rate of concomitant CIS (*n* = 52 [6.1%] vs. *n* = 107 [43%] BCG/EMDA-MMC and *n* = 35 [45.5%] RC, *p* < 0.001). There was no significant difference in recurrence and progression to muscle-invasive disease between the BCG-only and BCG/EMDA-MMC group (*n* = 336 [39.4%] and *n* = 102 [41%] recurrences and *n* = 158 [18.5%] and *n* = 53 [21.3%] progressions in the BCG and BCG/EMDA-MMC groups, respectively, *p* = 0.56 and *p* = 0.33). Similarly, there were no differences in time to recurrence (median: 16 [8–39 months]) and time to progression (median: 20 [9–36 months]) between the BCG and BCG/EMDA-MMC groups. Median follow-up time duration was significantly shorter in the upfront cystectomy group (37 months [IQR 26–63]) compared to the other groups (53 months [IQR 24–77] and 76 months [IQR 53–100], respectively, for BCG and BCG/EMDA-MMC, *p* < 0.001). The majority of patients were alive at the last available follow-up (*n* = 942 [80%] alive and *n* = 236 [20%] deceased), with no significant difference between the three groups (*p* = 0.28). Considering only deceased patients, the BCG/EMDA-MMC cohort had the longest period of follow-up before death (55 months [IQR 22–86] vs. 48 months [IQR 24–72] and 50 months [IQR 25–75] when compared to BCG only and upfront RC groups, *p* = 0.01).

### 3.2. Univariate Survival Analysis

#### 3.2.1. Kaplan–Meier Analysis for RFS

**Figure 1:**
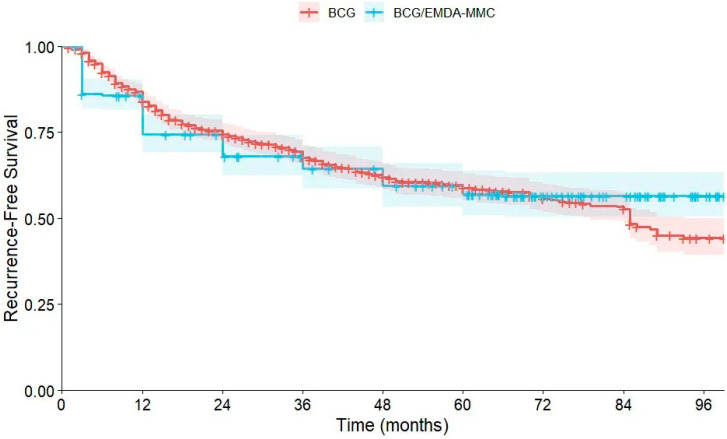
Kaplan–Meier analysis comparing RFS distributions between the BCG-only and BCG plus EMDA-MMC groups.

The univariate effect of the treatment group (BCG only vs. BCG/EMDA-MMC) on RFS was explored through a Kaplan–Meier survival analysis (Figure 1). By definition, intravesical recurrence cannot occur following radical cystectomy; therefore, these patients were excluded from the analysis. Mean survival time was 135.99 months (standard error [SE] = 11.99, 95% CI [Confidence Interval] 112.48–159.49]) for the BCG-only group and 96.78 months (SE = 4.63, 95% CI 88.03–105.53) for the BCG/EMDA-MMC group. The overall mean RFS across the two groups was 150.16 months (SE = 12.66, 95% CI 125.33–174.99). The Tarone–Ware test was the preferred method to calculate the equality in RFS distribution between the two groups, as it is more sensitive in capturing the differences during the middle follow-up period. Results showed no significant differences between the two groups (X^2^ = 0.46, *p* = 0.83). The similar results of additional tests (the Breslow test [*p* = 0.29] and log-rank test [*p* = 0.29]) confirmed that RFS was mostly similar across the entire follow-up period.

#### 3.2.2. Kaplan–Meier Analysis for PFS

**Figure 2:**
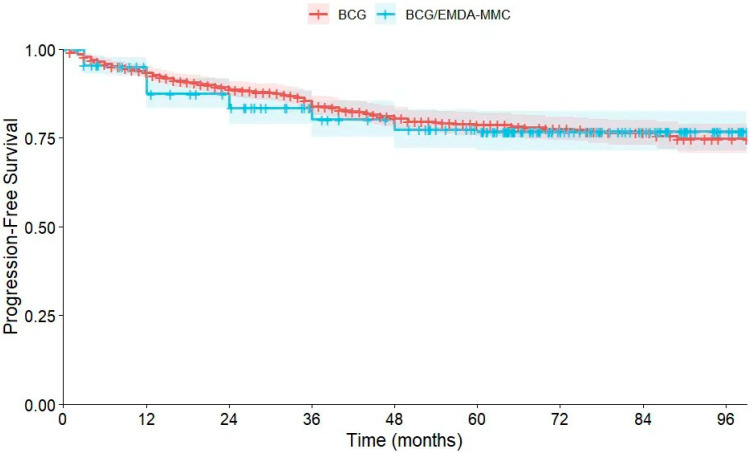
Kaplan–Meier analysis comparing PFS distributions between the BCG and BCG plus EMDA-MMC groups.

The Kaplan–Meier survival analysis comparing PFS distributions between the BCG and BCG/EMDA-MMC groups is shown in Figure 2. Progression was defined as progression to muscle-invasive disease, and thus patients undergoing upfront RC were not included in the PFS analysis. The mean survival times were 138.94 months (SE = 3.26, 95% CI 132.54–145.34) and 127.35 months (SE = 3.87, 95% CI 119.76–134.93), respectively, for the BCG and BCG/EMDA-MMC groups, with an overall mean survival time of 141.34 months (SE = 2.56, 95% CI 136.33–146.36). The log rank test was adopted to explore the equality of PFS distributions between treatment groups, with results showing no significant differences (X^2^ = 0.17, *p* = 0.89). Results of additional tests similarly confirm no significant differences between the two groups (*p* = 0.42 for Breslow and *p* = 0.64 for Tarone–Ware tests).

#### 3.2.3. Kaplan–Meier Analysis for OS

**Figure 3:**
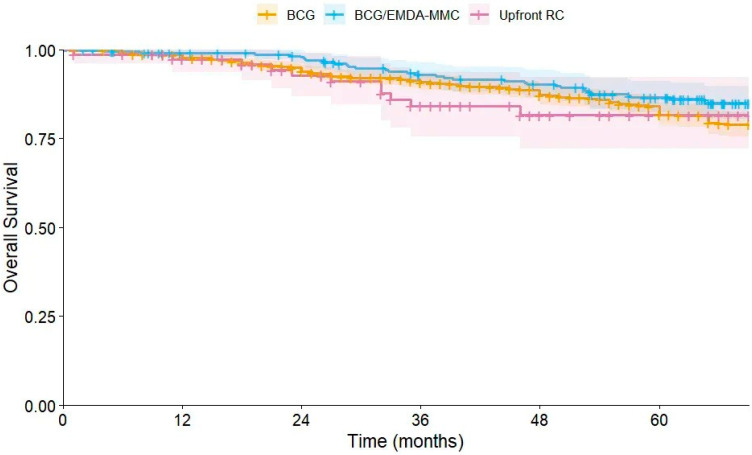
Kaplan–Meier analysis comparing OS distributions between the BCG, BCG plus EMDA-MMC, and upfront RC group.

Kaplan–Meier survival analysis was performed to evaluate the univariate effect of the treatment group on OS (Figure 3). Mean survival time was 131.16 months (SE 3.23, 95% CI 124.73–137.59) for the BCG group, 126.54 months (SE 3.7, 95% Ci 119.23–133.84) for the BCG/EMDA-MMC group, and 66.24 months (SE 2.42, 95% CI 61.49–70.99) for the upfront cystectomy group. Overall, the entire population demonstrated a mean survival time of 131.72 months (SE 2.8; 95% CI 126.21–137.23). The log-rank test showed no significant differences between the groups (X^2^ = 3, *p* value = 0.21). Similarly, the difference demonstrated by additional tests did not reach the level of significance (*p* = 0.08 for Breslow test and *p* = 0.10 for Tarone–Ware test).

### 3.3. Multivariable Cox Regression Models for RFS, PFS, and OS

Cox regression models exploring the multivariate effect of individual clinically relevant variables on RFS, PFS, and OS is shown in Table 2. NMIBC recurrence and progression to muscle-invasive disease were not influenced by the type of intravesical treatment (HR: 0.95, 95% CI 0.732–1.238, *p* = 0.71 for RFS and HR: 0.80, 95% CI 0.557–1.155, *p* = 0.24 for PFS, respectively for BCG/EMDA-MMC vs. BCG only). Similarly, none of the evaluated therapeutic approaches (BCG/EMDA-MMC vs. BCG only vs. upfront RC) was able to independently affect OS (HR: 1.00, 95% CI 0.701–1.424, *p* = 1.00 for BCG/EMDA-MMC vs. BCG only: HR: 1.13, 95% CI 0.591–2.143, *p* = 0.72 for upfront RC vs. BCG only; HR: 0.89, 95% CI 0.47–1.7, *p* = 0.73 for BCG/EMDA-MMC vs. upfront RC). Increasing age and a concomitant CIS were the only variables able to independently influence Recurrence times (HR: 1.03, 95% CI 1.015–1.036, *p* < 0.001 for age and HR: 1.39, 95% CI 1.05–1.85, *p* = 0.02 for concomitant CIS). A higher stage and a concomitant CIS significantly and independently reduced progression times (HR:1.47, 95% CI 1.073–2.009, *p* = 0.02 for stage and HR: 1.95, 95% CI 1.357–2.815, *p* < 0.001 for concomitant CIS). Conversely, performing a second resection demonstrated an independent favourable effect of PFS (HR: 0.72, 95% CI 0.544–0.945, *p* = 0.02). Regarding OS, beyond the inherent effect of increasing age (HR: 1.07, 95% CI 1.054–1.086, *p* < 0.001), higher tumour stage demonstrated a significant and independent negative impact (HR: 2.28, 95% CI 1.591–3.254, *p* < 0.00). 

## 4. Discussion

BCG treatment is by far the most adopted adjuvant treatment option for intermediate-, high-, and very high-risk NMIBC patients [17]. MMC intravesical administration has been intensively used as an adjuvant therapy [18]. It demonstrated higher tolerability and lower side effects when compared to BCG, with a more manageable administration protocol [19]. However, due to its demonstrated lower effectiveness and following the latest EAU recommendations, it is currently only adopted for low-risk and selected intermediate-risk patients [6]. Electromotive drug administration (EMDA) provides an active means of drug delivery, which employs electrical current to induce a directional and accelerated movement of the ionized drug towards the bladder epithelium [20]. Consequently, the EMDA device is reported to increase MMC penetration by four to seven times when compared to passive infusion [21]. EMDA-MMC administration, in combination with BCG instillation and delivered in a sequential fashion, showed promising results, in terms of improved recurrence-free interval and reduced progression rate [11,22]. However, since its first proposal nearly twenty years ago, the BCG/EMDA-MMC combination has rarely been employed. Accordingly, a recent systematic review and meta-analysis was able to include only two studies evaluating the efficacy and safety of the EMDA-MMC/BCG combination as a primary adjuvant treatment option for high-risk NMIBC [23]. Despite the promising results, the scarcity of data and methodological limitations of the available literature prevented definite conclusions about its efficacy. Upfront RC remains the sole treatment with the ability to drastically reduce the chances of recurrence [24]. It also offers a preferable choice for individuals who are unlikely to maintain consistent follow-up care, minimizing the requirement for invasive surveillance methods, particularly repeated cystoscopic examinations that may include tissue sampling [25]. However, the invasiveness of the procedure and the significant impact of RC and urinary diversion on patients’ urinary, sexual, and bowel function limit its applicability [26,27].

In the present study, we aimed to explore real-world comparisons of survival outcomes of high-risk and very high-risk NMIBC patients undergoing conservative treatment with TURBT followed by either BCG alone or in combination with EMDA-MMC and upfront RC. Age and gender distribution did not significantly differ between the three groups, and, as expected from a high- and very high-risk NMIBC cohort, the majority of patients had a high-grade Tumour. Patients who underwent treatment with BCG alone had demonstrated significantly lower rates of concomitant CIS. As this was not a randomized trial, it is likely that the presence of CIS, due to its proven negative impact on BCG responsiveness, recurrence, and progression rates, influenced MDT decision to opt for more aggressive treatment options, such as BCG/EMDA-MMC or upfront RC [28,29]. Nevertheless, there were no significant differences in recurrence and progression rates between the BCG and BCG/EMDA-MMC groups, and no significant difference in survival rates between the three groups. These results are in contrast with those reported by the original randomized controlled trial by Di Stasi and colleagues, who reported better outcomes for the EMDA-MMC/BCG combination group [11]. However, considering that our population only included high-risk and very high-risk NMIBC patients, with a median follow-up of 58 months, the resulting progression rate of 17.9% is significantly lower than that reported in current EAU guidelines. Moreover, our overall 37.2% recurrence rate is closer to that reported for the EMDA-MMC/BCG cohort of the original Di Stasi publication rather than the BCG-only cohort [11]. Regarding the survival times, the BCG/EMDA-MMC protocol showed no significant improvement in recurrence and progression times compared to the BCG-only cohort, but significantly longer overall survival time compared to the other two groups. Nonetheless, in multivariate analysis, the treatment group was not able to independently and significantly impact RFS, PFS, and OS, suggesting that the aforementioned differences are more likely to be attributed to concomitant variables, such as age, stage, grade, and CIS, rather than the treatment of choice.

Our study carries several limitations. Firstly, due to the retrospective nature of the study, important patient (comorbidities and number of previous TURBTs) and tumour (number of lesions, diameter, and presence of histological variants) characteristics were not available and therefore not measured within the population. The availability of the aforementioned characteristics could have pointed to a clearer pattern of preoperative selection with unpredictable effects on study results. For instance, a higher disease burden in terms of tumour size and multifocality may have led physicians to recommend radical or combination therapy, whereas the presence of multiple comorbidities might have influenced the choice to offer BCG alone. Accordingly, the proportion of high- and very high-risk patients may differ significantly between treatment cohorts, as it is not possible to retrospectively determine whether patients treated before the introduction of the up-dated EAU NMIBC risk stratification would have been classified as high- or very high-risk under the current model. Secondly, treatment cohorts were derived from different institutions, possibly increasing the risk of selection bias. This risk was further exacerbated by the inclusion of only pathologically confirmed NMIBC cases in the RC cohort and by the different re-TURBTs rates among treatment group. Thirdly, relevant information regarding treatment duration, compliance, and side effects was not available for review. Lastly, the different sample sizes of the explored groups, and particularly the relatively small number of included patients undergoing upfront RC, as well as the significantly different lengths of follow-up, could have masked significant differences between the groups.

## 5. Conclusions

Optimal management of high-risk and very high-risk NMIBC remains controversial. While the rationale behind bladder-sparing strategies lies in the initial stage of the disease, upfront RC is justified by the high recurrence rates and risk of progression, especially for very high-risk patients. In the present large multicentre retrospective study, we found survival outcomes to be influenced by preoperative and perioperative variables, such as age, stage, concomitant CIS, and re-TURBT. The treatment group (BCG, BCG/EMDA-MMC, and upfront RC), had, by contrast, no significant impact on RFS, PFS, and OS. Our results suggest no clear superiority of one strategy over the others within our cohort. On the contrary, the treatment of choice should rely on additional variables and, more importantly, on patients’ preferences balanced with oncologic risk and clinical judgement. In the future, large multicentre prospective studies are required to better shed light on this complex matter.

## Figures and Tables

**Table 1:** Baseline characteristics of the entire NMIBC cohort and stratified by treatment group.

	Total	BCG	BCG/EMDA-MMC	Upfront Cystectomy	*p*-Value
	1178	852	249	77	
**Age (years), median (IQR)**	71 (64–78)	71 (64–79)	69 (60–75)	71 (61–75)	0.21
**Gender**					0.22
Male	976 (82.8%)	705 (82.7%)	212 (85.1%)	59 (76.6%)	
Female	202 (17.1%)	147 (17.3%)	37 (14.9%)	18 (23.4%)	
**pT stage**					<0.001
pTa	390 (33.1%)	262 (30.8%)	113 (45.4%)	15 (19.5%)	
pT1	788 (66.9%)	590 (69.2%)	136 (54.6%)	62 (80.5%)	
**Grade**					<0.001
Low Grade	48 (4.1%)	23 (2.7%)	23 (9.2%)	2 (2.6%)	
High Grade	1130 (95.9%)	829 (97.3%)	226 (90.8%)	75 (97.4%)	
**Concomitant CIS**					<0.001
No	984 (83.5%)	800 (93.9%)	142 (57.0%)	42 (54.5%)	
Yes	194 (16.5%)	52 (6.1%)	107 (43.0%)	35 (45.5%)	
**Re-TURBT**					<0.001
No	558 (47.4%)	407 (47.8%)	136 (54.6%)	15 (19.5%)	
Yes	620 (52.6%)	445 (52.2%)	113 (45.4%)	62 (80.5%)	
**Recurrence**					0.56
No	663 (56.8%)	516 (60.6%)	147 (59.0%)		
Yes	438 (37.2%)	336 (39.4%)	102 (41.0%)		
**Time to recurrence, median (IQR)**	16 (8–39)	17 (8–42)	12 (3–27)		0.07
**Progression**					0.33
No	890 (75.6%)	694 (81.5%)	196 (78.7%)		
Yes	211 (17.9%)	158 (18.5%)	53 (21.3%)		
**Time to progression, median (IQR)**	20 (9–36)	21 (7–40)	12 (12–36)		0.14
**Alive at last follow-up**					0.28
No	236 (20.0%)	169 (19.8%)	56 (22.5%)	11 (14.3%)	
Yes	942 (80.0%)	683 (80.2%)	193 (77.5%)	66 (85.7%)	
**Time to death (months), median (IQR)**	48.5 (25–72)	48 (24–72)	55 (22–86)	50 (25–75)	0.01
**Follow-up (months), median (IQR)**	58 (27–85)	53 (24–77)	76 (53–100)	37 (26–63)	<0.001

**Abbreviations:** Re-TURBT: repeat transurethral resection of bladder tumour; BCG: Bacillus Calmette–Guérin; BCG/EMDA-MMC: Bacillus Calmette–Guérin/electromotive drug administration—mitomycin C; pT: pathologic stage; CIS: carcinoma in situ; NMIBC: non-muscle invasive bladder cancer.

**Table 2:** Cox regression models showing the multivariate effect of individual clinically relevant variables on RFS, PFS, and OS.

Characteristic	Recurrence-Free Survival			Progression-Free Survival			Overall Survival		
	HR	95% CI	*p* Value	HR	95% CI	*p* Value	HR	95% CI	*p* Value
Gender	0.98	0.760–1.257	0.86	0.96	0.666–1.379	0.82	1.34	0.979–1.838	0.07
Group (BCG plus EMDA-MM vs. BCG)	0.95	0.732–1.238	0.71	0.80	0.557–1.155	0.24	1.00	0.701–1.424	1.00
Group (Cystectomy vs. BCG)	-	-	-	-	-	-	1.13	0.591–2.143	0.72
Group (BCG plus EMDA-MM vs. Cystectomy)							0.89	0.47–1.7	0.73
Age	1.03	1.015–1.036	<0.001	1.01	0.995–1.023	0.20	1.07	1.054–1.086	<0.001
Stage	1.07	0.861–1.327	0.55	1.47	1.073–2.009	0.02	2.28	1.591–3.254	<0.001
Grade	1.02	0.647–1.606	0.93	1.03	0.545–1.963	0.92	1.01	0.546–1.872	0.97
Concomitant CIS	1.39	1.050–1.850	0.02	1.95	1.357–2.815	<0.001	1.27	0.870–1.846	0.22
Re-TURBT	0.84	0.695–1.020	0.08	0.72	0.544–0.945	0.02	0.87	0.667–1.129	0.29

**Abbreviations:** HR: hazard ratio; CI: confidence interval; Re-TURBT: repeat transurethral resection of bladder tumour; BCG: Bacillus Calmette–Guérin; BCG plus EMDA-MMC: Bacillus Calmette–Guérin plus electromotive drug administration—mitomycin C; CIS: carcinoma in situ.

## Data Availability

The data presented in this study are available on request from the corresponding author due to privacy restrictions.
